# Goblet cell adenocarcinoma: a case report and update regarding investigation and management

**DOI:** 10.1093/jscr/rjad215

**Published:** 2023-04-22

**Authors:** Laura C Deveson

**Affiliations:** Department of General Surgery, Eastern Health, Melbourne 3135, Australia

**Keywords:** Goblet Cell Adenocarcinoma, Appendix

## Abstract

This article describes the case of a patient who presented with an acute abdomen. Histopathology of the ruptured appendix identified Goblet Cell Adenocarcinoma. The biology of this rare tumour is now better understood, leading to updated best practise regarding investigation, staging and management.

## INTRODUCTION

Goblet Cell Adenocarcinoma (GCA) of the appendix is rare, therefore there is no consensus on best management. Recent advancements in the understanding of tumour biology of GCA has led to an update in terminology and guidance on how to best investigate, stage and manage the disease.

## CASE REPORT

An 81-year-old male presented to the Emergency Department with a 2 day history of abdominal pain with associated fever and anorexia. Past medical history included cardiac disease (severe aortic stenosis awaiting transcatheter aortic valve implantation, atrial fibrillation on apixaban, cardiac stent) and type two diabetes. He had no previous abdominal surgeries. He was septic on arrival; heart rate 162, temperature 41.2°C, blood pressure 111/73, respiratory rate 32. Examination revealed a distended abdomen with generalized tenderness. Investigations revealed white cell count 14.5, neutrophils 13.4 and C-reactive protein 86.9. Computed tomography (CT) of the abdomen and pelvis identified distended loops of small bowel in the right lower quadrant with adjacent free fluid but no gas. The patient underwent emergency laparotomy which identified a gangrenous, perforated appendix. This was resected and histopathology confirmed GCA involving multiple areas of the disrupted appendix including the apex and base. Depth of invasion extended to the subserosal fat (pT3). The case was reviewed at the Colorectal Multidisciplinary Meeting with a recommendation for consideration of right hemicolectomy once the patient has recovered.

## DISCUSSION

GCA of the appendix is a rare entity with an incidence of 0.05–0.3 per 100 000 per year [1]. GCA is characterized by the presence of adenocarcinoma as well as neuroendocrine carcinoma and has previously been described with varying terminology including goblet cell carcinoid or goblet cell carcinoma. These terms were revised in 2019 based on a better understanding of tumour biology and the recognition that the neuroendocrine component is minor, therefore, GCA is the preferred term [2].

Diagnosis is usually an incidental finding post appendicectomy and staging is via the TNM staging system. The T stage is based on the depth of invasion rather than size of the tumour, such is the case with adenocarcinoma. A CT chest/abdomen/pelvis then completes the staging.

There is limited evidence for further imaging with fluorodeoxyglucose positron emission tomography. Other imaging modalities such as octreotide scan, iodine 123 metaiodobenzylguanidine and GA-DOTATATE PET are usually negative. Tumour markers including carcinoembryonic antigen, Ca19.9 and Ca125 have been reported as raised in some cases, however, neuroendocrine tumour markers including chromogranin A and B are rarely raised. Appendiceal GCA is associated with synchronous or metachronous colonic malignancies, therefore, follow up colonoscopy is recommended [1].

Right hemicolectomy is the mainstay in surgical management for appendiceal GCA and results in a significant survival benefit for T3 and T4 tumours. For T1 and T2 tumours however there is no difference between appendicectomy and right hemicolectomy [3].

In conclusion, GCA should be investigated and staged similar to adenocarcinoma, thereby reducing the need for unnecessary investigations. We recommend discussion at a multidisciplinary meeting and consideration of completion right hemicolectomy particularly for T3 and T4 tumours.
